# Impact of Users' Attitudes Toward Anonymous Internet Interventions for Cannabis vs. Alcohol Use: A Secondary Analysis of Data From Two Clinical Trials

**DOI:** 10.3389/fpsyt.2021.730153

**Published:** 2021-09-27

**Authors:** Danilo Romero, Magnus Johansson, Ulric Hermansson, Philip Lindner

**Affiliations:** ^1^Centre for Psychiatry Research, Department of Clinical Neuroscience, Karolinska Institutet, & Region Stockholm Health Care Services, Region Stockholm, Stockholm, Sweden; ^2^Centre for Dependency Disorders, Region Stockholm, Stockholm, Sweden

**Keywords:** attitudes, internet interventions, latent subgroups, K-means (KM) clustering, alcohol, cannabis

## Abstract

**Background:** Numerous trials have demonstrated the efficacy of internet interventions targeting alcohol or cannabis use, yet a substantial proportion of users do not benefit from the format, warranting further research to identify moderators of treatment effects. Users' initial attitudes toward treatment is a potential moderator, yet no previous study has investigated users' attitudes in the context of internet interventions for addictive disorders.

**Method:** In this secondary analysis on two internet-based trials targeting harmful alcohol use (*n* = 1,169) and regular cannabis use (*n* = 303), respectively, we compared user groups' attitudes at the item level; explored within-group heterogeneity by submitting attitude scores to a k-means cluster analysis; and investigated whether latent subgroups in each user group moderated the treatment effects. Outcome models were run using generalized linear models with 10,000 bias-corrected bootstraps accounting for subject-level clustering.

**Results:** While substance groups and latent subgroups converged in enjoying the anonymity provided by the format, their interest toward treatment differed. Outcome analyses revealed a significant and negative time by subgroup effect on grams of cannabis consumed and screening test score (CAST), favoring the subgroup with positive treatment attitudes. There were not any significant effects of subgroup on alcohol consumption. Despite initial treatment reluctance, participants in the neutral subgroup decreased their cannabis use (gram) significantly when receiving the intervention vs. control.

**Conclusions:** This first, exploratory study revealed key differences between substance groups' attitudes, but more importantly that within-group heterogeneity appear to affect cannabis outcomes. Assessing attitudes could be key in patient-treatment matching, yet more research is needed.

## Introduction

Alcohol and cannabis are among the most used psychoactive substances globally (1). Every third user is likely to experience a transition from recreational use to addiction at some point in their life (2), entailing substantial psychosocial and monetary costs for the affected individual, significant others and society at large (3). Even though there are effective, evidence-based interventions only an estimated 19.8% with alcohol use disorder (AUD) and 13.2% with cannabis use disorder (CUD) will ever seek treatment (4, 5). Perceived and institutional barriers to seeking and entering treatment for alcohol use include treatment unavailability (6, 7), and attitudinal factors such as fear of stigma and shame (8–11) and finding traditional treatment services unattractive (10). In addition to these hinders, treatment-seeking for cannabis use is associated with particular challenges: many users view cannabis as an important part of their identity that demonstrates independence and free-thinking (12, 13). Congruently, a common top reported reason for not seeking cannabis treatment is the desire for self-reliance (14, 15). This likely contributes to the considerably low treatment uptake for individuals with CUD (14).

In addition to resolving many of the institutional barriers to disseminating evidence-based treatments at scale and at a low cost (16), internet interventions also have the potential to overcome many of the attitudinal barriers to treatment seeking: past research has revealed that service users (hereafter labeled users) experience internet interventions targeting alcohol use as accessible (6) and safekeeping privacy (6, 17). The interventions typically involve cognitive behavioral skill building exercises (identifying high-risk situations, craving management, etc.), formulated according to the principles of motivational interviewing, with or without therapist guidance through asynchronous text communication. It has been proposed that internet interventions may be particularly suitable for satisfying cannabis-dependent individuals' desire for self-reliance (15), yet it has not been empirically examined. Indeed, little is known how users perceive internet interventions for regular cannabis use, and it is unclear how their views converge and differ from those of users of alcohol internet interventions. Increased knowledge about the user groups' views toward features and components of internet-based treatments, as well as factors that facilitate treatment-seeking, would be of great utility in developing transdiagnostic designs, tailoring the interventions to each target group, and validly translating insights from each respective treatment group.

A growing number of trials support the efficacy of internet interventions in reducing alcohol and cannabis consumption (16–19), yet a substantial proportion of users do no benefit from the interventions and the effect sizes for interventions targeting regular cannabis use tend to be small (18). A potential moderator of treatment effects that has been overlooked in the extant literature is the attitudes that users hold toward internet interventions. Attitudes, referring to the degree to which a person evaluates a behavior or an entity as favorable or not (20, 21), is a key concept in health and social psychology that is hypothesized to be a determinant of intention and behavior change—a claim that has gained empirical support (22). The concept of attitudes has been adopted to explain acceptance toward emerging digital technologies, such in the original Technology Acceptance Model (23), and has during the last decade been recognized to be a specifically important research target for the field of internet interventions (24). Several studies have investigated the general public's (25, 26), stakeholders' (27), clinicians' (28–30) and different patient populations' (31, 32) attitudes toward internet interventions, yet only a handful of studies have investigated whether users' attitudes at treatment entry affect outcome.

A recent study on a 1-month transdiagnostic intervention targeting anxiety symptoms found that initial attitudes moderated the treatment effect with a small effect size relative to care as usual, such that users with positive initial attitudes who received the intervention reported greater symptom decrease over time (33). Similarly, it has been demonstrated that the effect of an online intervention for depression was contingent on initial attitudes, also in favor of positive initial attitudes with a small effect size (34). Importantly, subscale analysis revealed that participants with greater negative baseline-attitudes as measured by the scale “technologization threat,” which covers reluctance to participating in computerized treatment instead of in-person contact, experienced increased depression symptom over time compared to the control group (34). Although not relying on direct moderation analyses, another study on depression showed that positive initial attitudes toward online treatment predicted better outcomes and that users with greater negative baseline attitudes were more likely to discontinue their online treatment (35).

The extant literature thus suggests that baseline attitudes are associated with differential treatment effects. Apart from some subscale analyses, previous research has used unidimensional sum scores which may not capture the full complexity of attitudes. Specifically, this may disregard attitudinal ambivalence: holding both positive and negative appraisals toward an attitude object (36). Moreover, considering that internet trials typically have lower threshold for entry (e.g., liberal inclusion criteria, minimal waiting time) than traditional services, and recruit participants beyond professional help systems, it is likely that the trials include a more heterogeneous sample with respect to attitudes. Such heterogeneity can be explored with analytical approaches that attempt to cluster individuals into latent subgroups that share similar patterns of attributes (37), in this specific case attitudes, going beyond aggregated scores by identifying specific constellations of responses. To our knowledge, no previous study has explored the impact of users' attitudes toward internet interventions targeting any addictive disorder or whether latent subgroups of users, derived from patterns of attitudes, moderate outcomes.

The aim of the current study was three-fold: (a) to examine difference in specific attitudes toward internet interventions among users of programs for alcohol vs. cannabis use; (b) to explore latent subgroups based on attitudes in each of the two groups of users; and (c), to explore whether such subgroups moderated the respective treatment effect.

## Method

### Sample and Procedure

This study is a secondary analysis of data from two randomized controlled trials of internet interventions for harmful alcohol use or dependence (38) and regular cannabis use (39), respectively. See each respective publication for details on participant recruitment, procedure, treatments and full outcomes reporting. In brief, the trial targeting alcohol use (*N* = 1,169) compared a 12-week therapist-guided intervention and self-help intervention with an information-only control (1:1:1) at 3- and 6-months follow-ups. Users of the therapist-led intervention had greater decrease in alcohol consumption at 3 months compared to the control, although other time by group effects were not significant (38). The trial targeting regular cannabis use (*N* = 303) compared a guided self-help intervention with a wait-list control (WLC) in a ratio of (1:1), showing no significant time by group effects on cannabis use at the 3-month follow-up, yet such effects were revealed in the subset of participants that had not sought other professional help during the trial (39). In both trials, all participants were asked to rate their attitudes toward internet interventions (see full description of measure below) before entering the program, of which *n* = 1,037 + 256 participants answered the form, implying a coverage rate of 89% and 84% in the alcohol and cannabis program, respectively. The current study uses these ratings, along with baseline variables and outcome measures from each trial. Missing data in attitude items were handled using either case-wise omission (if percentage missing data exceeded 10%) or imputed using the case-wise median. Following this criterion, 4 of 1,293 subjects were omitted and 47 values imputed. The final sample consisted of *n* = 1,289 participants: *n* = 1,037 users of the alcohol program and *n* = 252 users of the cannabis program. Both the alcohol (2014/1758-31/2) and cannabis trials (2014/1374-31/5) were approved by the Swedish Ethical Review Authority, as were the specific analyses described herein (2021-02322). All participants provided informed consent.

### Measures

To measure users' attitudes toward web-based programs, both studies included a newly developed 33-item form (see Supplementary Material) that covered three aspects of believed importance: reasons for choosing internet-based treatment (7 items), views on features of the program (16 items) and views on the specific treatment components (10 items). The form included three additional items in the alcohol trial that were excluded to enable user-group comparisons. All items were rated on a 11-point scale ranging from 0-10, with verbal anchors at 0 (“Completely unimportant, does not need to be included”) and 10 (“Very important, I would not consider using the service without this feature”). The items were identical across the studies except for terms referring to each user group. For presentation purposes herein, substance-specific terms were substituted with the generic term “substance” and items were translated from Swedish by DR, followed by independent back-translation by PL to ensure validity. The questionnaire showed satisfactory internal consistency in each substance group, as measured by McDonald's Omega (Alcohol: ω = 0.849, 95% Wald CI = 0.832-−0.867; Cannabis: ω = 0.852, 95% Wald CI = 0.817 −0.887) (40). Readiness to change substance use was measured in both trials using the 100-point Visual Analog Scale (41).

Primary outcome measures in the alcohol and cannabis trials were past-week number of standard drinks and past-week number of days of cannabis non-use, respectively. Both these measures were extracted from data that was collected using the Timeline Follow-Back method (42). Secondary outcome measures included past-week binge-drinking (the original count response format was dichotomized in this study due to severe overdispersion: ϕ = 2.367), the Swedish 10-item version of the Alcohol Use Disorder Identification Test (43), past-week consumption of cannabis in gram, and the Swedish 12-item version of the Cannabis Abuse Screening Test (CAST) (44), in the respective trials, as well as self-reported alcohol or cannabis use disorder criteria (45).

### Analyses

All analyses were performed using the R (version 4.1.0) statistical environment (46). Data was analyzed in three steps, corresponding to the three research questions. First, means and parametric 95% confidence intervals were calculated for each item, and then compared between the alcohol and cannabis groups. Second, to explore latent subgroups in each respective group, raw item-level scores were submitted to a k-means cluster analysis for each group separately. K-means is a learning algorithm that partitions a group of observations into clusters, given a specified parameter *k* (47, 48). The algorithm starts by establishing *k* centers randomly, and then alternates between assigning each observation to the closest cluster center and repositioning the cluster centers to the temporary mean of the affiliated observations, in a process that iterates until the centers become fixed. We set the distance metric to *Manhattan* (L1 norm), as it has been suggested to perform better than the default Euclidean metric (L2 norm) in high-dimensional data sets that features 20 or more variables (49). A central issue in clustering is specifying the parameter *k* (50). Numerous indices exist and these often generate competing suggestions. To not rely on a single index, we evaluated possible cluster solutions (ranging 2–10 clusters) using 26 fit indices provided by the NbClust R package (51). We selected the cluster solution that was suggested by most indices. Next, we used item-level means and parametric 95% confidence intervals to explore attitudinal differences between the subgroups. To assess the effects of user groups and latent subgroups on treatment attitudes, above and beyond the effect of age and readiness to change, respectively, we ran separate multiple linear regressions at the item level.

Finally, having explored latent subgroups in each respective group, we investigated whether attitude cluster moderated treatment effects in each substance group, by including cluster affiliation as a predictor with three-way interactions in generalized linear models that additionally featured time and treatment predictors, along with full-factorial interaction terms and 2 × 2 × 3-factorial interaction terms for the cannabis-use and alcohol-use group, respectively. As there were no significant changes in outcomes between 3- and 6-months follow up in the alcohol-use group as well as high attrition (38), the follow-up time-points were collapsed to retain power. These models were run separately for each substance group using the relatively assumption-free cluster-bootstrap generalized linear modeling approach with subject-level clustering and 10 000 bias-corrected bootstraps (52).

## Results

### Comparison Between User Groups

**Figure 1:**
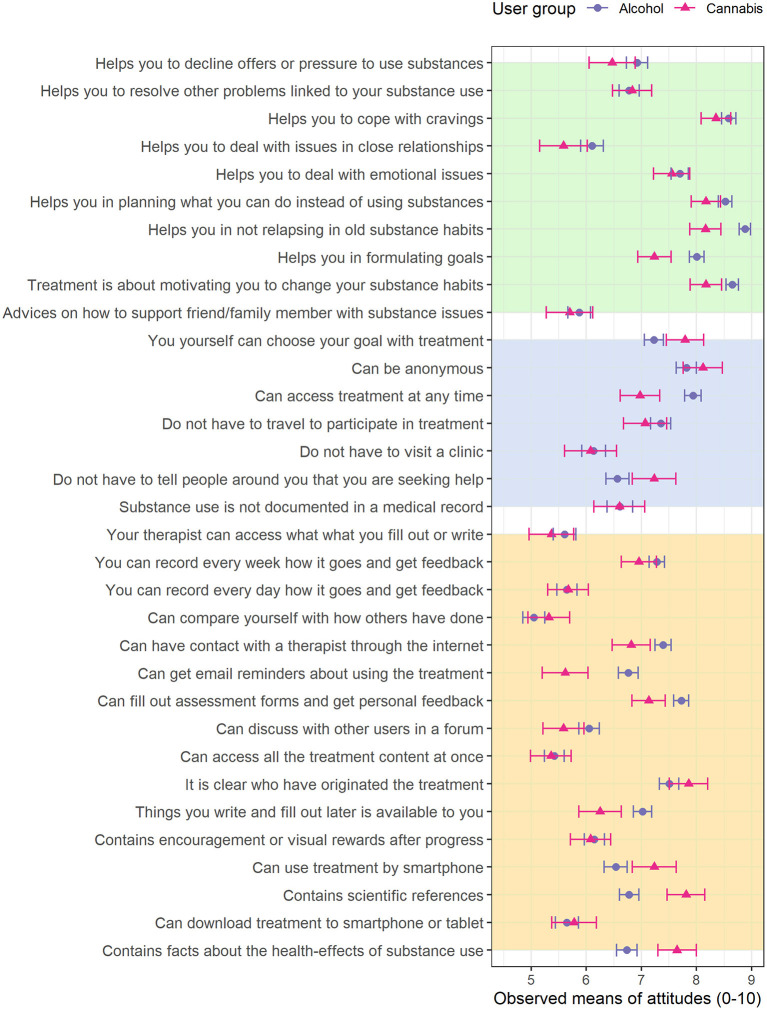
Observed means at the item level, grouped by substance group. The error-bars represent the parametric 95% confidence interval (CI). Background colors represent question category (Yellow: treatment features; Blue: reasons for seeking internet-based treatment; Green: treatment components).

Substance groups differed significantly in mean scores on more than a third of the items (13 of 33 items). On items covering possible reasons for seeking internet-based treatment, both substance groups saw great value in anonymity, but the aspect of not having to disclose treatment-seeking to other people was on average higher valued in subjects with CUD, as was the autonomy-emphasizing aspect that the program allow the user to set his or her own goal, whereas the alcohol-use group had a higher preference for being able to access treatment at any time. In general, both substance groups endorsed the treatment components that reinforce intra-individual skills, such as dealing with cravings, whereas treatment ingredients that target inter-individual skills (e.g., support in dealing with issues in close relationships) were considered less important. The core treatment components of goal-setting support, relapse-prevention and motivation-elicitation were higher valued by the alcohol-use group. The cannabis-use group reported no higher preference for any of the treatment components compared to the alcohol-use group. Regarding the format of the program, the alcohol-use group considered it more important to be able to have contact with a therapist through the internet and receive personal feedback. In contrast, the cannabis-use group had a higher preference for the informational content of the program, i.e., that the program included facts about how cannabis affects health and included references to scientific sources. See Figure 1 for item-level mean scores and 95% confidence intervals. Attitudinal differences between substance groups were significant above and beyond the effect of age in 9 of the 13 items that originally displayed between-group differences in means (see Supplementary Table 1).

### Latent Subgroups

**Figure 2:**
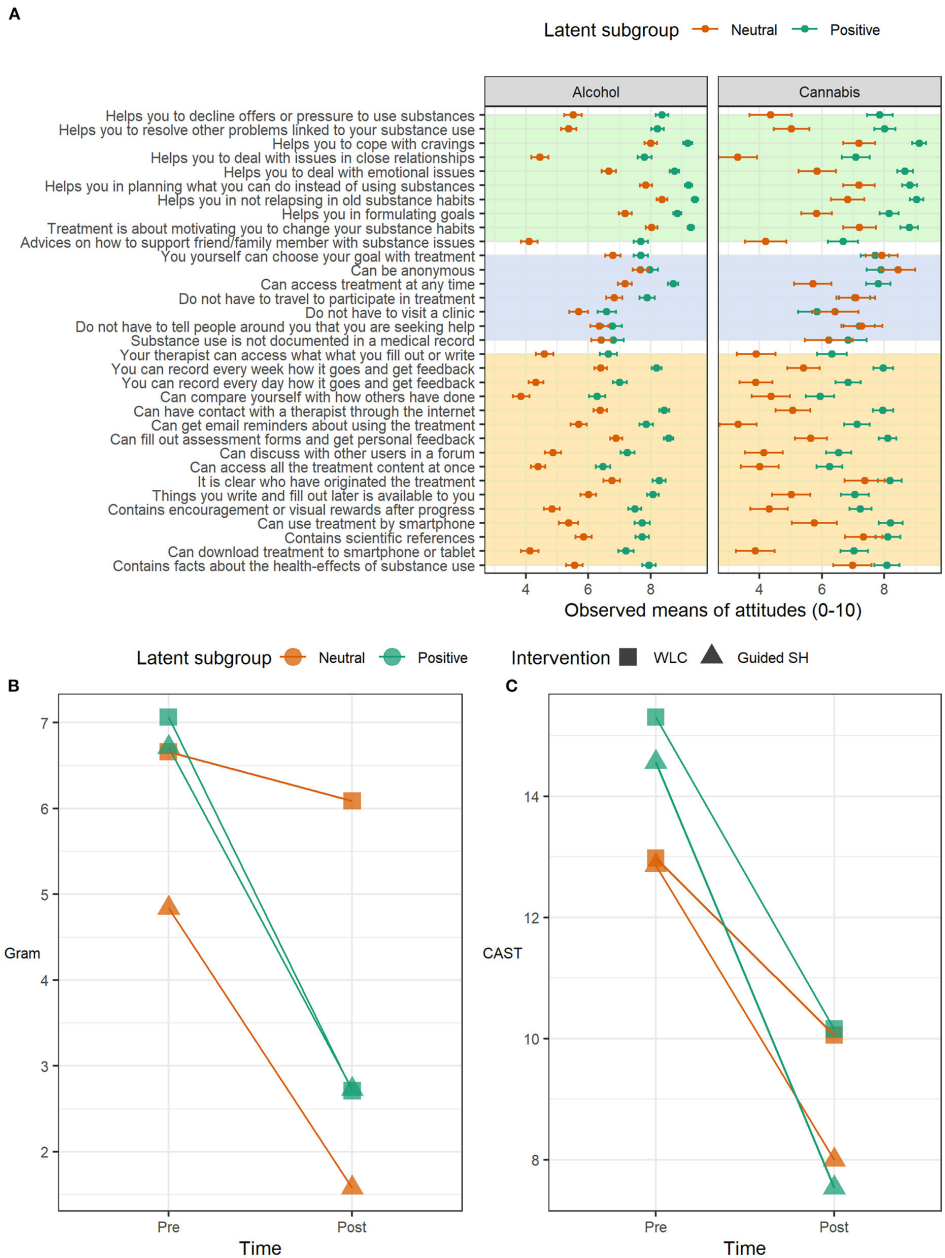
**(A)** Observed means at the item level, grouped by latent subgroup within each respective user group. The error-bars represent the parametric 95% confidence interval (CI). Background colors represent question category (Yellow: treatment features; Blue: reasons for seeking internet-based treatment; Green: treatment components). The lower panel contains outcome plots for observed means in cannabis consumption over time grouped by intervention (WLC, Wait-list control; SH, Self-help) and latent subgroup. **(B)** Self-reported grams used during the previous seven days; **(C)** 12-item version of the Cannabis Abuse Screening Test.

The optimal cluster solution for the attitude ratings was *k* = 2, favored by 10 of 26 indices in both user groups (see Supplementary Table 2 for model fit indices and Supplementary Figure 1 for the two graphical indices). Latent subgroups differed significantly on all but a few items in the alcohol-use group (30 of 33 items) and on most items in the cannabis-use group (25 of 33 items). The first subgroup gave consistently lower attitude ratings than the second subgroup (and vice versa) across all item-level differences in both substance groups (Figure 2). Grand mean differences between latent subgroups motivated the labels of “Neutral attitudes” for the first subgroup (Alcohol: *M* = 6.01, *N* = 525; Cannabis: *M* = 5.65, *N* = 100) and “Positive attitudes” for the second subgroup (Alcohol: *M* = 7.89, *N* = 512; Cannabis: *M* = 7.56, *N* = 152) in both user groups. The latent subgroups exhibited similar constellations of responses across substance groups: the neutral and positive subgroups in each substance group differed in appraisal of the treatments' components and features but converged in endorsing anonymity aspects. However, a closer comparison of the response patterns that constituted the neutral subgroup in each substance group revealed differences: the neutral subgroup in the alcohol trial seemed to view the treatment with overall uninterest, only approving of the anonymity aspects, whereas the neutral subgroup in the cannabis-use group additionally approved of goal-setting autonomy, availability and transparency aspects, suggesting a basic interest in engaging with the material but not necessarily for treatment purpose. Item-level mean differences between latent subgroups remained significant in most cases (Alcohol trial: 27 of 30 mean differences; Cannabis trial: 18 of 25 mean differences) when accounting for readiness to change (Supplementary Table 3).

### Associations With Outcomes

#### Internet Interventions for Harmful Alcohol Use

There was a main effect of time across all outcomes. There were not any significant effects of attitude subgroup on any outcome: neither main effects nor two- or three-ways interactions effects with time and/or treatment predictors (Table 1).

**Table 1:** Outcome model parameters of the alcohol program.

	**Standard Units**	**Presence of binge**	**AUDIT score**	**Number of DSM-5 alcohol**
	**(past week)**	**drinking (past week)**		**use disorder criteria**
Type	Negative Binomial	Binomial	Gaussian	Gaussian
Intercept	4.01 (3.767 to 4.28)^*^	4.41 (3.286 to 5.478)^*^	28.3 (26.522 to 30.264)^*^	8.56 (7.846 to 9.314)^*^
Time	−0.71 (−0.931 to −0.489)^*^	– 2.16 (−2.767 to −1.536)^*^	−7.17 (−8.67 to −5.767)^*^	−1.82 (−2.364 to −1.301)^*^
GSH	−0.14 (−0.481 to 0.203)	−0.37 (−1.911 to 1.137)	2.07 (−0.623 to 4.767)	0.28 (−0.773 to 1.317)
USH	−0.01 (−0.349 to 0.324)	0.17 (−1.347 to 1.692)	2.19 (−0.448 to 4.867)	0.65 (−0.365 to 1.664)
Subgroup	−0.21 (−0.56 to 0.127)	0.09 (−1.509 to 1.672)	2.08 (−0.587 to 4.69)	0.62 (−0.413 to 1.658)
Time x GSH	0.07 (−0.223 to 0.358)	0.31 (−0.552 to 1.146)	−1.77 (−3.842 to 0.319)	−0.31 (−1.063 to 0.474)
Time x USH	<0.00 (−0.305 to 0.302)	−0.08 (−0.92 to 0.76)	−1.74 (−3.85 to 0.333)	−0.56 (−1.341 to 0.232)
Time x subgroup	0.12 (−0.162 to 0.418)	0.08 (−0.795 to 0.941)	−0.44 (−2.482 to 1.602)	0.02 (−0.727 to 0.788)
USH x subgroup	0.16 (−0.363 to 0.664)	−0.37 (−2.519 to 1.791)	0.36 (−3.451 to 4.064)	0.14 (−1.351 to 1.615)
GSH x subgroup	0.42 (−0.056 to 0.896)	2.29 (−0.269 to 4.759)	1.18 (−2.597 to 4.918)	1.11 (−0.332 to 2.573)
Time x GSH x subgroup	−0.39 (−0.791 to 0.006)	−1.36 (−2.663 to 0.006)	−1.43 (−4.244 to 1.445)	−0.86 (−1.947 to 0.213)
Time x USH x subgroup	−0.16 (−0.584 to 0.292)	0.01 (−1.17 to 1.199)	−0.6 (−3.56 to 2.288)	−0.04 (−1.135 to 1.078)

*Unstandardized regression coefficients and 95% confidence intervals (CI) using bias corrected and accelerated cluster bootstrap intervals. GSH, Guided self-help; USH, Unguided self-help; AUDIT, Alcohol Use Disorders Identification Test; DSM-5, Diagnostic and Statistical Manual of Mental Disorders, Fifth Edition*.

* *Statistically significant*.

#### Guided Self-Help for Regular Cannabis Use

There were significant positive main effects of subgroup on cannabis consumption (grams), CAST and diagnostic criteria, implying higher baseline severity among users with positive treatment attitudes (Table 2). Significant negative time by subgroup effects on gram and CAST revealed that the positive subgroup experienced superior improvement over time relative to the neutral subgroup. Average changes in cannabis consumption (gram) and CAST over time grouped by treatment and latent subgroup are shown Figure 2. Time by subgroup interaction effects on gram and CAST-score remained significant (Gram: *B* = −0.73, CI = −1.464, −0.196; CAST: *B* = −2.46, CI = −4.815, −0.226) in *post-hoc* analysis excluding participants who had sought other professional help. *Post-hoc* analysis conducted on the subgroup with neutral attitudes revealed a statistically significant time by treatment interaction effect on the secondary outcome gram (*B* = −1.06, CI = −1.67, −0.266). For full report of *post-hoc* model parameters, see Supplementary Table 4. There was not any significant main effect of positive subgroup (*B* = 0.10, SE = 0.38, *p* = −0.788) or interaction effect between positive subgroup and WLC (*B* = 0.73, SE = 0.54, *p* = 0.175) on loss to follow up.

**Table 2:** Outcome model parameters of the cannabis program.

	**Number of days without**	**Gram cannabis**	**CAST score**	**Number of DSM-5**
	**cannabis use (past week)**	**(past week)**		**cannabis use disorder criteria**
Type	Negative Binomial	Negative Binomial	Gaussian	Gaussian
Intercept	−0.59 (−1.435 to 0.157)	1.99 (1.646 to 2.243)^*^	15.9 (14.089 to 17.641)^*^	9.52 (8.525 to 10.587)^*^
Time	0.69 (0.258 to 1.161)^*^	−0.11 (−0.342 to 0.167)	−2.92 (−4.267 to −1.696)^*^	−1.92 (−2.719 to −1.249)^*^
GSH	0.09 (−1.018 to 1.213)	0.75 (−0.136 to 1.428)	1.82 (−1.523 to 5.457)	0.29 (−1.717 to 2.235)
Subgroup	−0.03 (−1.067 to 1.032)	0.93 (0.31 to 1.729)^*^	4.55 (1.843 to 7.428)^*^	1.76 (0.314 to 3.228)^*^
Time × GSH	0.34 (−0.293 to 0.941)	−1.06 (−1.652 to −0.226)^*^	−1.94 (−4.582 to 0.512)	−0.77 (−2.192 to 0.704)
Time × subgroup	0.3 (−0.317 to 0.881)	−0.87 (−1.493 to −0.359)^*^	−2.22 (−4.409 to −0.119)^*^	−0.59 (−1.753 to 0.537)
GSH × subgroup	−0.1 (−1.537 to 1.331)	−0.85 (−1.983 to 0.437)	−0.68 (−5.606 to 4.034)	0.15 (−2.422 to 2.8)
Time × GSH × subgroup	−0.3 (−1.095 to 0.525)	1.11 (−0.029 to 2.02)	0.06 (−3.636 to 3.89)	0.04 (−2.001 to 2.043)

*Unstandardized regression coefficients and 95% confidence intervals (CI) using bias corrected and accelerated cluster bootstrap intervals. GSH, Guided self-help; CAST, Cannabis Abuse Screening Test; DSM-5, Diagnostic and Statistical Manual of Mental Disorders, Fifth Edition*.

* *Statistically significant*.

## Discussion

The present study explored and compared users' attitudes toward anonymous internet interventions targeting alcohol use and cannabis use. While the substance groups generally converged in enjoying the unique aspects of internet interventions (e.g., anonymity, no transportation required), our results suggest that users of the alcohol program give higher priority to individual treatment components, such as relapse prevention, whereas users of the cannabis program to a greater extent value the informational and autonomy-emphasizing features of the program. Our data highlight the need to explore within-group heterogeneity in users' attitudes. We showed that the interest toward the cannabis program as an informational service was restricted to a subgroup of users rather than the overall sample, and that there was indeed an unobserved subgroup of users that put high value on the treatment components, signaling an intention to engage in treatment. Importantly, this interest toward treatment predicted decreased cannabis consumption over time, independent of treatment allocation. Latent subgroups in the alcohol trial were characterized by positive and neutral attitudes, respectively, but within-group heterogeneity was not significantly associated with treatment outcome.

In line with prior research on users' attitudes toward internet interventions for other disorders (33–35), positive attitudes predicted superior outcome for users of the cannabis trial. This suggests that attitudes toward treatment to some degree have a generalizable and transdiagnostic relevance in predicting outcome, yet the attitude-outcome association was not significant for users of the alcohol intervention. Noteworthy, as concerns the outcomes standard drinks and binge-drinking, the bias-corrected confidence intervals for the three-way interaction effects only slightly overlapped the null. Without implying that a larger sample size would prove these effects to be significant (53)—indeed the sample size in the alcohol trial was more than four times as large as the cannabis sample—further observations in independent samples are needed to fully reject the hypothesis that initial attitudes moderate alcohol treatment outcomes. Increased understanding about whether the attitude-outcome association differs across patient populations could be important for concentrating research efforts, and selectively translating findings into clinical practice. A possible explanation for the non-significant results could be that within-group differences in attitudes among users of alcohol interventions are not optimally represented as stemming from distinct subgroups of users, but rather are unidimensional. Our finding that subgroups in the alcohol trial differed in *overall* appraisal of the program, whereas subgroups in the cannabis trial demonstrated more complex response patterns—shared a basic interest in the program while differing in interest toward treatment—appear to support such an interpretation, yet it should be considered that the trials had unequal sample sizes and thereby unequal statistical power to detect subgroup differences.

Although there is a growing literature that positive attitudes moderate outcome, little is known about the symptom trajectories of users with negative or neutral attitudes. Our *post-hoc* analysis demonstrated that even the subgroup with neutral attitudes in the cannabis trial—the subgroup that initially had a basic interest in the material but little interest in using it for treatment purposes—benefitted from the intervention relative to WLC, as shown by a significantly greater decrease in consumption (grams of cannabis). If this finding can be replicated in independent samples, it would not only confirm the potential of guided self-help in attracting people who are reluctant to engage in treatment, but also its capability in eliciting motivation and inducing behavior change despite initial treatment reluctance. Considering the notable treatment gap among individuals with CUD, and the desire for self-reliance being the top attitudinal barrier to treatment-seeking, this would have large clinical and public health value. Importantly, individuals in the subgroup with neutral attitudes that received treatment had lower baseline consumption compared to WLC, indicating lower levels of addiction severity. It may be that guided self-help is particularly beneficial for individuals in the early stages of addiction progression that are reluctant to engage in treatment. These findings must however be interpreted with caution as they emerged from *post-hoc* analysis.

To our surprise, the attitude-outcome association was not restricted to treatment but also present in WLC in the cannabis trial. Importantly, participants in both conditions were blinded to when the other arm would receive treatment. As there neither was a main effect of subgroup nor a subgroup by WLC interaction effect on loss to follow up, the observed wait-list improvement is unlikely to spring from attrition bias. Further, the time by subgroup interaction effects remained significant when excluding subjects that had sought other help, thus, external interventions were not the source of wait-list improvement. However, it is possible that time by subgroup interaction effect was due to omitted variable bias: in specific, it is well-documented in the addiction field that novel, negative consequences related to the substance use simultaneously can increase the motivation to seek help (54–56) and induce sudden behavior change. Considering that heightened willingness to seek help may positively influence the attitudes toward the requested treatment, this bias is likely augmented for the positive subgroup. Interestingly though, the positive subgroup had high baseline levels of cannabis consumption, even significantly greater than the neutral subgroup (on three of four outcome measures), suggesting that treatment-seeking preceded behavior change. In view of these findings, we hypothesize that mere participation in baseline measurements had a positive influence on the subgroup of users with positive attitudes. More specifically, baseline measurements could have triggered increased awareness and self-monitoring (57), eliciting behavior change even in absence of the intervention (58). Positive assessment effects has been suggested to be a cause of wait-list improvement in psychological trials (57) and has also previously been observed in the addiction literature (59, 60). If these attitude-outcome findings in the cannabis trial replicate they could have substantial implications for patient-treatment matching: high-intensity therapist resources could be allocated to the subgroup with neutral attitudes, whereas the subgroup with initial positive attitudes could be offered automated or low-intensity therapist-led interventions, potentially optimizing the outcome of internet interventions targeting regular cannabis use.

In summary, our exploratory analyses revealed that there are key differences between substance groups' attitudes toward treatment but more importantly that within-group heterogeneity specifically matters for cannabis outcomes. Considering the low treatment utilization in addictive disorders, and the potential of internet interventions in overcoming some of the barriers to treatment, there is a need for further research for whom it works, with attitudes potentially being a moderator of large clinical value.

### Limitations

This first study on users' attitudes toward internet interventions for addictive disorders has some limitations. First, although we evaluated possible cluster solutions using 26 fit indices, current applications of the k-means algorithm do not allow testing for and rejecting sample homogeneity (*k* = 1) in multivariate data. Second, the instrument used to measure attitudes was custom-made and had not been independently validated, nor were comprehensive psychometric analyses included in the current study. Internal consistency was however satisfactory, and the analytical approach focused on the item rather than scale level, rendering many standard psychometric evaluation indices superfluous. Third, the current study was not designed to evaluate the impact of a full set of potential differences (e.g., socio-economic status) that are likely to exists between cannabis and alcohol users; importantly, regardless of indirect attribution, our findings nonetheless have implications for the treatment of these groups as they present. Moreover, we did include age and readiness to change as covariates in some models. Future research should examine further constructs that potentially confounds between- and within-group differences in attitudes. Fourth, the survey underlying these secondary analyses was not mandatory, possibly introducing self-selection bias as users with greater positive attitudes could have been more inclined to answer the form. However, importantly, the survey had high coverage rates in both trials and within-group heterogeneity in attitudes was great regardless, indicating no sampling bias.

## Data Availability Statement

The data that support the findings of this study are available from the corresponding author upon reasonable request.

## Ethics Statement

The studies involving human participants were reviewed and approved by Swedish Ethical Review Authority. The patients/participants provided their written informed consent to participate in this study.

## Author Contributions

DR, MJ, and PL: designed the study. MJ and UH: collected data, made substantial contributions to analysis, or interpretation of findings. DR and PL: analyses and drafted the manuscript. All authors contributed in revising the manuscript for important intellectual content and approved the final version to be published.

## Funding

DR was funded by Region Stockholm and the Swedish Research Council for Health, Working Life and Welfare (2019-01642). The alcohol and cannabis trials were supported by separate grants.

## Conflict of Interest

The authors declare that the research was conducted in the absence of any commercial or financial relationships that could be construed as a potential conflict of interest.

## Publisher's Note

All claims expressed in this article are solely those of the authors and do not necessarily represent those of their affiliated organizations, or those of the publisher, the editors and the reviewers. Any product that may be evaluated in this article, or claim that may be made by its manufacturer, is not guaranteed or endorsed by the publisher.
